# Improving antenatal care in prisons

**DOI:** 10.2471/BLT.14.151282

**Published:** 2015-08-31

**Authors:** Molly Skerker, Nathaniel Dickey, Dana Schonberg, Ross MacDonald, Homer Venters

**Affiliations:** a City University of New York, Hunter College of Sociology, New York, United States of America (USA).; b The New York City Department of Health and Mental Hygiene, Bureau of Correctional Health Services, 42-09 28th St, Queens, NY, 11101, USA.; c Montefiore Medical Center, Albert Einstein College of Medicine, New York, USA.

Significant progress is being made to improve the outcomes of pregnancy and childbirth in many countries. In many low- and middle-income countries, including Ethiopia, Bangladesh, Bolivia (the Plurinational State of), Myanmar and Pakistan, great strides have been made in significantly reducing maternal mortality.^1^ However, the estimated 24 000–60 000 women who are pregnant and incarcerated worldwide often lack access to antenatal care at the same level as that available in their communities.^2^ Despite clear international standards that mandate equivalent care for people in prison, pregnant women in these settings face significant barriers to adequate antenatal care.^3^^–^^5^ The needs of pregnant women are often overlooked in prisons designed to house men – who comprise most of the world’s prison population of over 10.1 million people.^3^^,^^6^ As the World Health Organization’s Member States consider the post-2015 agenda for maternal health, this vulnerable and hidden cohort of women should not be forgotten.

The first challenge to provision of adequate antenatal care in prisons is the location of correctional health systems within ministries of the interior or other security authorities. These institutions have large structures dedicated to maintaining security and health care providers tend to be marginalized in such settings.

Many women, including those who are pregnant, are held in settings without access to toilets or water for washing or bathing and sleeping quarters often require sleeping on hard surfaces without a mattress or pillow.^7^ The use of shackles and other restraints create significant discomfort and stress for women throughout pregnancy and especially during labour.^8^ Most correctional settings lack dedicated obstetricians or skilled antenatal specialists. Because the basic medical services available to prisoners often do not include physical examination and reproductive history, women who need referral are not always identified. Women who can afford to pay for care may be the only ones to receive antenatal services in prison.

A second challenge to the provision of antenatal care for women in prisons is the high rates of pre-existing conditions that affect maternal health, including sexually transmitted infections, hepatitis, mental illness and substance abuse.^9^^–^^13^ Consequently, pregnant women in prison often need coordinated antenatal, medical and behavioural health services.^14^^,^^15^ Women infected with human immunodeficiency virus (HIV) and/or affected by opiate dependency are routinely denied safe, effective treatments for these conditions when pregnant, often due to a lack of educated health staff.

A third barrier to adequate antenatal care is the pervasiveness of sexual assault and other human rights abuses in correctional settings.^16^^,^^17^ Women endure high rates of physical and sexual assault in prison.^18^ Female prisoners may be forced into coercive sexual relationships to receive basic medical services, family visits or even food.^16^ Another concern is the practice of so-called virginity testing, used in some settings for women entering prison.^19^ This practice amounts to sexual assault, even if performed by medical staff at the direction of security staff. The resulting physical and psychological trauma directly harms women and contributes to distrust of whatever antenatal care is available, especially when health staff are seen to be aligned with the security services.

A fourth issue is the lack of reliable data. Most prison health systems are separated from the ministry of health or public health department and do not report pregnancies, live births or complications in the population they serve. This lack of information is a barrier to improving – or tracking changes in – conditions.

We propose three responses to these challenges. First, the same reporting metrics used in public health agencies should be applied to prisons. Similar strategies have been adopted for reporting of deaths in custody and the development of risk reduction policies.^20^ Basic information includes rates and causes of death and injuries, hospital transfers and the prevalence of chronic disease.

Second, national policies designed to improve the reporting of sexual abuse and other human rights abuses should include women in prisons.^21^^,^^22^

Third, successful programmes to reduce maternal mortality should be implemented in prisons. Two approaches that have been successful in Pakistan include prenatal health education in facilitator-led women's groups and training traditional birth attendants in infection control and emergency obstetric care.^23^ These types of interventions are a cost-effective means to improving maternal outcomes and could help integrate prison health services with those provided to the community.^24^^,^^25^

Improving antenatal care in prisons is important for improving maternal outcomes in all countries. New strategies that have emerged from discussions of the post-2015 development agenda include forging new partnerships and identifying and integrating vulnerable populations.^26^
